# A late appearance case of TEK-related mucocutaneous venous malformation treated by Nd:Yag laser

**DOI:** 10.1016/j.jdcr.2026.03.052

**Published:** 2026-04-03

**Authors:** Marine Mercipinetti, Paul Kuentz, Eulalie Laude-Pagniez, Clémence Boucher, Théo Brochet

**Affiliations:** a Department of Dermatology, Amiens-Picardie University Medical Center, Amiens, France; b Université Marie and Louis Pasteur University, Besançon Universitty Medical Center, TRANSLAD Hospital-University Federation, Oncobiology Genetics Bioinformatics, Besançon, France; c Department of Dermatology, Valenciennes Medical Center, Valenciennes, France; d Department of Dermatology, Groupe Hospitaliser du Sud de l’Oise (GHPSO), Creil, France

**Keywords:** cutaneomucosal venous malformation, Nd:Yag laser, TEK-related venous malformations

## Introduction

Venous malformations are congenital abnormalities in the development of blood vessels that may be discovered late in life as they evolve over time. These lesions are characterized by their multifocal nature, bluish appearance, and slight compressibility. They manifest as irregularly sized lesions on the skin and mucous membranes. These malformations are characterized by slow-flow patterns. From a histological perspective, venous malformations are characterized by the presence of large, tortuous vessels. These conditions may manifest sporadically or within familial clusters. Mucocutaneous venous malformations (VMCM) are genetically transmitted and account for 1% of venous malformations.^1^

VMCM have been associated with a mutation in the TEK gene, which is responsible for encoding the TIE2 tyrosine kinase receptor, which is specific to vascular endothelial cells. This autosomal dominant condition is characterized by an elevated TIE2 function, leading to accelerated vascular proliferation through the PI3K/AKT/mTOR pathway.^2^ The most prevalent of these is the germline mutation c.2545C>T; p(Arg849Trp), which is also known as R849W. However, it should be noted that numerous other mutations are possible and are being identified as research on the TEK gene progresses.^3^^,^^4^ Phenotypic penetrance, age of onset, and severity vary among carriers of the mutation. This phenomenon appears to be attributable to the action of a paralogous mechanism, involving a germline mutation followed by a secondary somatic mutation in the second allele of the same gene. This results in the complete abolition of the normal function of the gene.^3^, ^4^, ^5^ The identification of this second-hit mechanism in VMCM gave rise to the hypothesis that a comparable mechanism might also underlie sporadic venous malformations, which are more prevalent.^3^

## Observation

**Fig 1:**
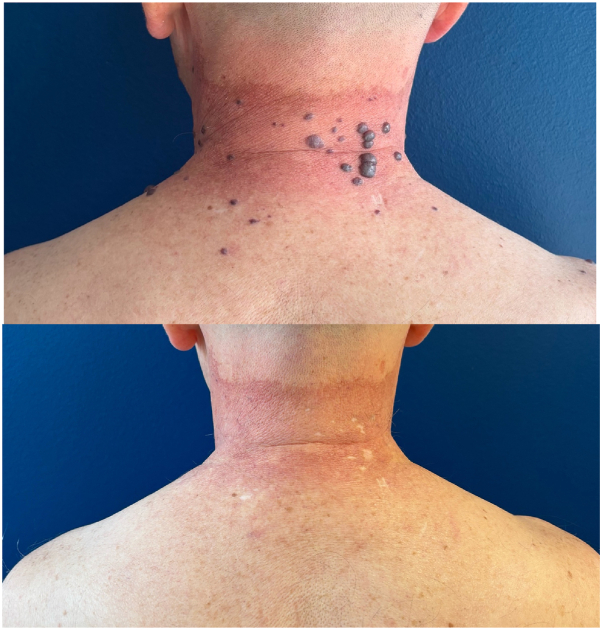
Posterior cervical venous malformations before and after Nd:YAG laser treatment.

**Fig 2:**
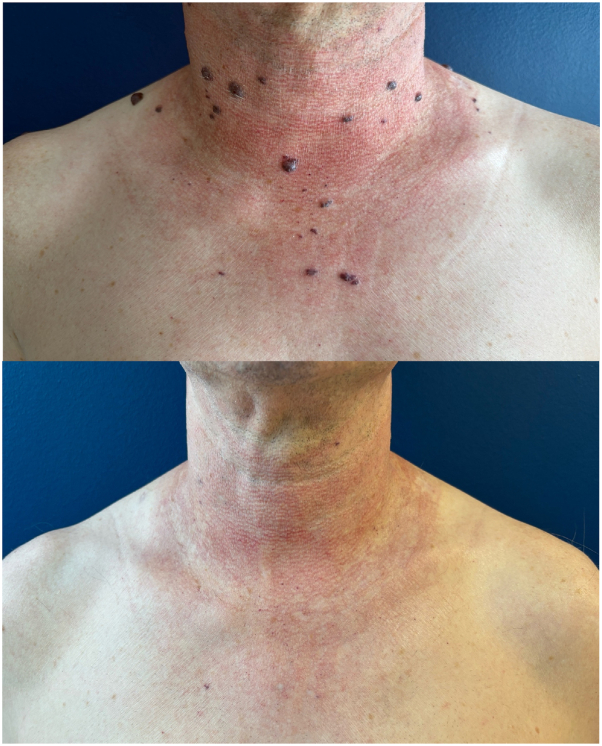
Anterior cervical venous malformations before and after Nd:YAG laser treatment.

**Fig 3:**
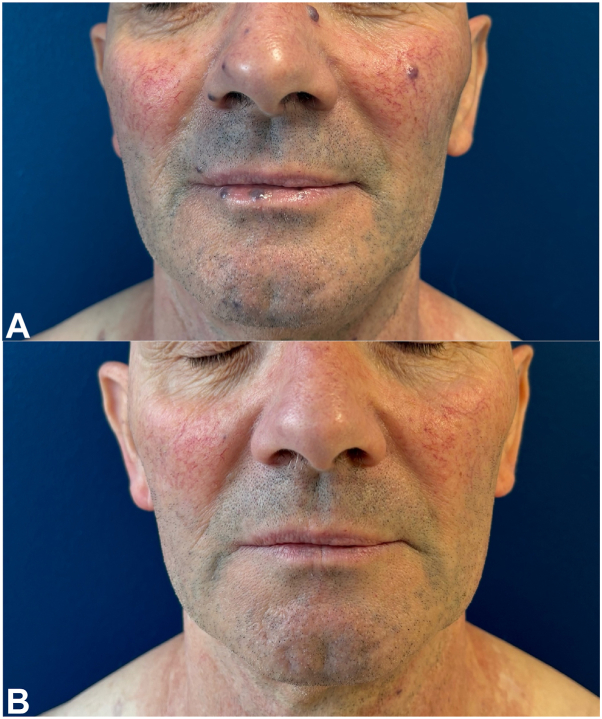
Venous malformations of the face and lips before (A) and after (B) Nd:YAG laser treatment.

A 64-year-old patient sought consultation with a dermatologist regarding multiple papular and nodular erythematous-violaceous lesions that were palpable and diminished upon pressure with a glass slide. The lesions were located on the anterior and posterior neck, face, and lips and had been developing for approximately 15 years. The patient’s medical history included prior craniotomy for subcutaneous meningocele and noninsulin-dependent diabetes, with ongoing treatment involving metformin, sitagliptin, and acetylsalicylic acid. The primary hypothesis of this study was that VMCM were the underlying cause. The patient’s heterozygous twin brother exhibited similar lesions, which also appeared around the age of 50. However, due to logistical constraints, the brother could not be examined. The biopsy revealed a vascular lesion consisting primarily of dilated and tortuous veins, with walls that exhibited variability in thickness, ranging from thin to muscular, and the thickness of the muscle varied accordingly. The vascular walls expressed CD34 but not D2-40. Somatic analysis by NGS panel of one of these lesions revealed a mutation in exon 17 of the TEK gene, C.2740C>T; p(LEU914Phe), which is referred to as L914F. The patient exhibited symptoms consistent with functional discomfort. Treatment was administered using a 1064-nm long-pulse Nd:YAG laser. Following 4 sessions with a spot diameter of 3 mm, a pulse duration of 30 ms, and a fluence of 150 J/cm^2^, with integrated cooling without stacking, all lesions were effectively treated in a single impact (Figs 1, 2 and 3).

## Discussion

Our case is noteworthy due to the presence of the recurrent L914F variant, a postzygotic missense variant located in exon 17 of the TEK gene, which is typically associated with sporadic unifocal venous malformations (without a second hit necessarily required in this form)^6^ but was considered to be the cause of the disease in our case. Sporadic mosaic mutations have not been demonstrated to be vertically transmissible or associated with a mutation in a sibling. The mutation identified (L914F) has not been previously documented in VMCM or sporadic multifocal venous malformations, and to date, no de novo form of VMCM has been reported.^7^ However, the patient had a heterozygous twin brother who exhibited the same lesions, suggesting a familial form of the condition. The lesions were not present in his 2 children, and he did not know his parents. A negative family history alone does not preclude a diagnosis given the possibility of different phenotypic penetrance depending on the individual (explained by the need for a second hit), the possibility of death before the onset of symptoms, or the late onset of symptoms. The absence of a family history cannot be determined without molecular genetic testing (10% of patients with a TEK gene mutation will not have lesions)^7^ and no genetic testing was performed on our patient’s relatives.

There are no clear recommendations for the management of venous malformations. Decisions are based on consensus conferences established in particular by the European expert group on vascular anomalies (VASCERN VASCA working group).^8^ The standard treatment modalities for VMCM include Nd:YAG laser therapy, percutaneous sclerotherapy (employing detergents, microfoam, bleomycin, absolute ethanol, or radio-opaque ethylcellulose-ethanol), and surgical excision.^1^^,^^9^ The employment of vascular lasers facilitates targeted, precise treatment without the necessity for invasive procedures, thereby mitigating the potential for adverse effects. However, it is imperative to use a cooling mechanism to prevent burns to the epidermis during treatment. The wavelength and power delivered by a laser are contingent upon the size, topography, color, and depth of the vascular lesion. The target chromophore is hemoglobin. Three primary types of vascular lasers are currently available for treatment: the flashlamp-pumped pulsed dye laser, which is indicated for small, superficial, pink-colored lesions; the Nd:YAG-KTP laser, which is indicated for larger lesions; and the long-pulse Nd:YAG laser, which is indicated for even deeper and larger lesions. For the venous malformations presented by the patient, the long-pulse Nd:YAG laser, which uses a wavelength of 1064 nm, was necessary to penetrate deeper into the dermis and treat the large vessels. The efficacy of the vascular laser is optimal when the lesions are diminutive, recent, and not located on the extremities. It is important to note that these treatment procedures are rarely curative, as relapses are inevitable. The extent of the lesions can also be a limiting factor.^10^ The elucidation of the genetic mechanisms implicated facilitates the development of targeted therapeutic interventions.^5^ The most extensively studied molecule to date is sirolimus, an mTOR inhibitor that blocks the PI3K/AKT/mTOR pathway, which is overactive in VMCM. Trials have also been conducted for rapamycin and everolimus. The role of these targeted therapies in the treatment of venous malformations remains to be determined.^7^, ^8^, ^9^

## Conflicts of interest

None disclosed.

## References

[1] Dompmartin A., Vikkula M., Boon L.M. (2010). Venous malformation: update on aetiopathogenesis, diagnosis and management. Phlebology.

[2] Vikkula M., Boon L.M., Iii K.L.C. (1996). Vascular dysmorphogenesis caused by an activating mutation in the receptor tyrosine kinase TIE2. Cell.

[3] Limaye N., Wouters V., Uebelhoer M. (2009). Somatic mutations in angiopoietin receptor gene TEK cause solitary and multiple sporadic venous malformations. Nat Genet.

[4] Wouters V., Limaye N., Uebelhoer M. (2010). Hereditary cutaneomucosal venous malformations are caused by TIE2 mutations with widely variable hyper-phosphorylating effects. Eur J Hum Genet.

[5] Van Damme A., Seront E., Dekeuleneer V., Boon L.M., Vikkula M. (2020). New and emerging targeted therapies for vascular malformations. Am J Clin Dermatol.

[6] Soblet J., Kangas J., Nätynki M. (2017). Blue rubber bleb nevus (BRBN) syndrome is caused by somatic TEK (TIE2) mutations. J Invest Dermatol.

[7] Seront E., Boon L.M., Vikkula M., Adam M.P., Feldman J., Mirzaa G.M. (1993). GeneReviews® [Internet].

[8] Dompmartin A., Baselga E., Boon L.M. (2023). The VASCERN-VASCA Working Group diagnostic and management pathways for venous malformations. J Vasc Anom.

[9] Queisser A., Seront E., Boon L.M., Vikkula M. (2021). Genetic basis and therapies for vascular anomalies. Circ Res.

[10] Bencini P.L., Tourlaki A., De Giorgi V., Galimberti M. (2012). Laser use for cutaneous vascular alterations of cosmetic interest: lasers for cutaneous vascular lesions. Dermatol Ther.

